# High platelet adrenergic activity and concomitant activation of the pituitary/medullar axis as alarming laboratory parameters in ACS survivors—the STRESS-AMI study

**DOI:** 10.3389/fcvm.2024.1338066

**Published:** 2024-02-21

**Authors:** Zalán Gulyás, Zsófia Horváth, László Hajtman, Andrea Kovács, László Kohut, István Kósa, Emese Tóth-Zsámboki, Róbert Gábor Kiss

**Affiliations:** ^1^ Department of Cardiology, Central Hospital of Northern Pest—Military Hospital, Budapest, Hungary; ^2^ Cardiac Rehabilitation Institute, Central Hospital of Northern Pest—Military Hospital, Balatonfüred, Hungary

**Keywords:** acute coronary syndromes, complex cardiac rehabilitation, platelet aggregation, salivary cortisol, epinephrine-induced platelet aggregation, secondary prevention

## Abstract

**Introduction:**

Kinetics of stress-related biological parameters were determined in acute coronary syndrome (ACS) patients undergoing complex cardiovascular rehabilitation.

**Methods:**

We determined platelet functionality in the absence/presence of a selective alpha-2 adrenergic receptor inhibitor, atipemazole parallel with salivary cortisol levels at enrolment, and at 3- and 12-months follow-up in 75 ACS patients with percutaneous coronary intervention.

**Results:**

Pharmacological/non-pharmacological secondary prevention methods have been efficiently applied. Baseline aggregometry indicated platelet hyperactivity, decreasing gradually and being significantly reduced late, at 12 months (*p* < 0.05). Cortisol levels followed similar kinetics (*p* < 0.05). Baseline epinephrine-induced aggregations (EIA) significantly correlated with most of the other platelet agonists, even at subsequent time-points. Patients with upper-quartile EIA at enrolment (EIA-UQ) had significantly higher ADP- and collagen-induced aggregations at enrolment, at 3- and 12-months follow-up as well, indicating that high adrenergic response in the acute phase is accompanied by general platelet hyperactivity and predicts sustained platelet activation. In the EIA-UQ group higher cardiac biomarker release, elevated C-reactive protein and cortisol levels, and lower baseline left ventricular ejection fraction were detected.

Atipemazole significantly reduced platelet aggregation induced by several platelet agonists, being most potent and comparable to full *in vitro* P2Y_12_ inhibition on collagen-induced aggregations (*p* < 0.05), indicating that catecholamines might serve as promt/long-term modulators of platelet function.

**Discussion:**

Despite effective CCR programme and dual antiplatelet therapy, prolonged activation of sympathetic neuroendocrine system and general platelet hyperactivity can be detected up to one year in ACS patients with high adrenergic platelet activity. Moreover, initial high adrenergic activity is accompanied by clinical parameters associated to increased cardiovascular risk, therefore early identification of these patients might support complex optimal long-term therapy.

## 1 Introduction

Cardiovascular mortality and underlying ischaemic heart disease is the leading cause of death worldwide (1). Majority of the atherothrombotic factors contributing to acute coronary syndromes (ACS) are modifiable and are a consequence of genetic factors, concomitant diseases and inappropriate lifestyle (2). In secondary prevention, a cardiac rehabilitation program improves the quality of life after the acute coronary event and reduces the risk of recurrent cardiovascular events (3). Previous study has shown that a complex multidisciplinary cardiac rehabilitation (CCR) program achieves definite reduction in cardiovascular risk and improves clinical factors, including platelet function as well (4). The optimal level of platelet inhibition plays a pivotal role in both pharmacological and non-pharmacological therapy after ACS and percutaneous coronary intervention (PCI) (5). High residual platelet reactivity (HRPR) has consistently been associated with recurrence of ischemic cardiac events and linked to increased cardiovascular mortality (6). Newer, more potent antiplatelet drugs (e.g., ticagrelor, prasugrel) significantly reduce on-treatment hyperactivity via more effective inhibition of the platelet P2Y_12_ ADP receptor (7, 8). Generally, a delicate balance between antiplatelet efficacy and individual bleeding risk provides the best clinical outcome. Hence, a second-generation less effective thienopyridine, clopidogrel is still widely used despite its unpredictable metabolism due to genetics polymorphism, comorbidities and drug interactions (9). Unfortunately, optimal level of platelet inhibition changes over time as well, as atherothrombotic burden is generally thought to be higher in the acute phase (10). Therefore, actual level of platelet inhibition on dual treatment still represents a clinically relevant but challenging factor during both the acute and chronic stage of coronary disease.

Pathophysiology of the increased platelet reactivity throughout the acute phase of coronary syndromes is not fully understood. There is increasing evidence that psychological stress contributes to cardiovascular disease (11–13). In response to psychological stress, catecholamines release from adrenal medulla and activate the sympathetic nervous system. Epinephrine—by binding to alpha-2-adrenergic receptors on platelets surface—activates platelets *in vitro* and induces platelet aggregation. In healthy humans, pathological concentrations of epinephrine are capable of recruiting platelets into the circulation and induce microaggregation and adhesion (14). It was proposed that catecholamine-induced platelet activation might be the link between stress and cardiovascular disease (15). Previously, atipemazole, a selective alpha-2-adrenergic receptor inhibitor was shown to fully inhibit epinephrine-induced platelet aggregation in stable coronary heart disease patients (16). Although it might provide insight on the contribution of platelet adrenergic receptor activation to the pathomechanism of acute coronary syndromes, the effect of alpha-2 adrenergic receptor inhibition on platelet aggregation in ACS patients is not known.

Acute or chronic stress is followed by a stimulation of the hypothalamus-pituitary-adrenal (HPA) axis resulting in the release of glucocorticoids, mainly cortisol from adrenal cortex (17). Cortisol has several heterogeneous effects on metabolism, immune- and cardiovascular system which help to adapt to external demands (18). Excessive or permanent release of cortisol promotes other pathological conditions (e.g., hypertension, glucose intolerance, hyperlipidaemia) linked to cardiovascular disease (19). Additionally, in the acute phase of coronary disease, studies show a correlation between higher cortisol levels and larger myocardial infarction size (20, 21), arrhythmias and higher in-hospital mortality in acute coronary syndrome (22, 23). Cortisol levels could be measured in hair, urine, blood or saliva. The unbound active fraction of cortisol is about 10% of the total plasma content, which can diffuse into the saliva and therefore could represent a non-invasive stress indicator (24).

Currently there are no comprehensive, parallel data concerning the release, kinetics and association of salivary cortisol and alpha-adrenergic platelet function in patients undergoing percutaneous coronary intervention and complex cardiovascular rehabilitation in the acute and chronic phase of acute coronary syndromes. Similarly, there is a lack of data on the contribution of adrenergic platelet activation to general on-treatment platelet hyperactivity. Therefore, the aim of this study was to examine the kinetics of sympathetic neuroendocrine activation by determining salivary cortisol levels parallel with general and specific, catecholamine-related platelet function in ACS patients in the acute and chronic phase, up to one year follow-up.

## 2 Materials and methods

### 2.1 Patients and study design

In this prospective, single-center study we enrolled 80 patients diagnosed with ACS undergoing primary or urgent PCI within a 5-months study period. The diagnosis of acute coronary syndrome was established according to the Universal Definition of Myocardial Infarction (25). Subsequently, the main inclusion criteria were the acceptance of an early CCR program and the attendance at scheduled follow-ups. Exclusion criteria included active malignancy, chronic renal dysfunction (serum creatinine above 170 μmol/L) or liver failure (ALAT or serum bilirubin above threefold of upper limit of normal), immunology disease or the use of glucocorticoids. Patients physically unable to participate in the rehabilitation program were excluded as well. Written informed consent was obtained from all eligible patients; the study complied with the Declaration of Helsinki and was approved by the local ethical committee, the regional Scientific and Research Ethics Committee (approval number is 602-2/2019/EÜIG) and the National Public Health Centre. Clinical data were registered, routine laboratory blood sample, platelet function and salivary cortisol levels were assessed at enrolment (84–96 h after hospital admission), as well as at 3- and 12-month follow-ups.

### 2.2 Complex cardiovascular rehabilitation program

Patients participated in a 3-week in-hospital CCR program within 30 days of the index ACS. The CCR program was composed of four principal elements: dietary interventions, physical activity, stress-management and cardiovascular education. Diet and physical activity were based on guidelines for the prevention of cardiovascular disease (26). Stress-management was facilitated through thematic support group session and individualised relaxation therapy (e.g., imagination technics, meditation, breathing therapy, muscle relaxation). The program was financed via the European Structural and Investment Funds (ESIFs) as the GINOP-2.1.1-15-2016-01076 project. It is important to emphasise that the patients did not receive any financial compensation or funding.

### 2.3 Platelet aggregometry

Platelet function was measured by Born light transmission aggregometry (Carat TX4 Aggregometer, Carat Diagnostics, Budapest, Hungary) at enrolment (84–96 h after hospital admission) and at 3-month and 12-month follow up. Platelet rich and poor plasma were separated from citrate-anticoagulated blood by centrifugation (180 g and 1800 g for 10 min, respectively). Platelet agonists were 1 and 2 μg/ml collagen (COLL, Takeda, Austria), 2.0 μg/ml epinephrine (EPI, Richter-Gedeon Ltd., Hungary), 0.5 μg/ml arachidonic-acid (AA, Sigma-Aldrich Ltd., Hungary) and to assess effectiveness of P2Y_12_ receptor inhibition 1.25, 5 and 10 μM adenosine diphosphate (ADP, Sigma-Aldrich Ltd., Hungary). In order to assess the effect of full *in vitro* P2Y_12_ and alpha-2 adrenergic platelet receptor inhibition, platelet rich plasma samples (300 μl) were preincubated with antagonists for 30 s; cangrelor, a specific, competitive platelet P2Y_12_ receptor antagonist (1 μM, The Medicines Company, Parsippany, NJ, USA) and atipemazol, a specific alpha-2 adrenergic receptor antagonist (2 μM, Antisedan, Pfizer Animal Health, Extron, PA, USA) were used. Measurement time was 7 min with 1,000 /min stirring at 37°C; maximal aggregation was determined in percentage.

### 2.4 Salivary cortisol collection and measurements

Salivary cortisol samples were collected at enrolment and at 3 and 12 months after the acute coronary event by swabbing from the oral mucosa. In order to avoid effects of the diurnal cortisol variation, all samples were taken between 8 and 10 am., even during scheduled control visits. Patients were instructed to fast, omit caffeine, and avoid smoking for a minimum of 3 h prior to sample collection. Samples were collected by the same personnel in a dedicated examination room, 5 min peace and quiet was mandatory before the sampling. Salivary samples were stored at 8°C before being transported to the external laboratory for processing.

Salivary cortisol levels were measured by standardised, automated electro- chemiluminescence immunoassay method (SYNLAB Hungary Ltd.).

### 2.5 Statistical analysis

Statistical analyses were performed using Statistica 8.0. software (Stat Soft. Inc., Tulsa, USA). Continuous parameters were tested for normal distribution by using the Shapiro–Wilk's W test. Normally distributed parameters are given as mean and standard error of the mean (SEM), parameters with non-normal distributions were summarized as median and interquartile ranges (IQR). Since most of the variables were non-Gaussian, non-parametric tests were applied. Differences between independent subgroups were evaluated by Mann–Whitney *U* tests. For repeated measures, the Wilcoxon signed-rank test was used. Categorical variables were compared with Fisher's exact test. Strength and direction of association between two ranked variables was measured by Spearman's rank correlation.

Required patient sample size was calculated based on previous Born aggregometry data (4). In case of 2 μg/ml collagen, 5 μM ADP and 2.0 μg/ml epinephrine the required sample size was calculated to be 30, 57 and 120 at 3 months, respectively (*α* = 0.05, *β *= 0.1). All the statistical analyses were two-tailed, and level of significance was *p* < 0.05. Patients with missing data or incomplete records were excluded from analysis.

## 3 Results

### 3.1 Patient baseline demographic data and results of the multidisciplinary cardiac rehabilitation program

The voluntary participation in the CCR program and the two subsequent scheduled visits were successfully completed by 75 individuals, indicating that these patients were committed to an active lifestyle change after an episode of acute coronary syndrome. Baseline demographic data are presented in Table 1; Supplementary Tables S1, S2.

**Table 1:** Clinical data and demographic parameters of the complete patient population and the two subgroups with epinephrine induced platelet aggregations in the lower and the upper quartile.

Demographic data	Total cohort (*n* = 75)	Epinephrine induced aggregations in the LQ (*n* = 19)	Epinephrine induced aggregations in the UQ (*n* = 19)	*p* =
Male/female, *n* (%)	63/12 (84/16)	17/2 (89/11)	17/2 (89/11)	1.00 / 1.00
Age, years	56.9 ± 8.8	55.1 ± 6.4	55.9 ± 10.1	0.44
BMI on admission, (kg/m^2^)	27.5 (25.2–32.1)	27.1 (25.3–33.3)	27.7 (24.9–33.8)	1.00
BMI at 12 months, (kg/m^2^)	26.6 (24.1–30.4)	25.8 (24.6–30.0)	28.0 (24.2–37.0)	0.34
Hypertension, *n* (%)	43 (57)	11 (58)	11 (58)	1.00
Systolic BP on admission, (Hgmm)	135.9 ± 19.9	138.3 ± 23.4	138.8 ± 14.7	0.96
Diastolic BP on admission, (Hgmm)	85.3 ± 12.9	80.6 ± 15.0	85.8 ± 11.2	0.66
Diabetes mellitus, *n* (%)	15 (20)	2 (11)	5 (26)	0.42
Hyperlipidemia, *n* (%)	36 (48)	8 (42)	12 (63)	0.58
Hyperuricemia, *n* (%)	9 (12)	3 (16)	2 (11)	1.00
Previous CAD, *n* (%)	21 (28)	5 (26)	4 (21)	1.00
Previous AMI/PCI, *n* (%)	6/10 (8/13)	2/3 (11/16)	0/1 (0/5)	0.49 / 0.61
Previous stroke/TIA, *n* (%)	2/3 (3/4)	1/0 (5/0)	1/0 (5/0)	1.00 / 1.00
PAD, *n* (%)	2 (3)	1 (5)	1 (5)	1.00
Thyroid disease, *n* (%)	8 (11)	3 (16)	1 (5)	0.61
Smoking previously, *n* (%)	17 (22)	3 (16)	6 (32)	0.47
Current smoker, *n* (%)	32 (42)	13 (68)	6 (32)	0.26
Smoking at 12 months, *n* (%)	17 (25)	7 (44)	3 (16)	0.31
Regular exercise on admission, *n* (%)	29 (39)	8 (42)	9 (47)	1.00
Regular exercise at 12 months, *n* (%)	44 (80)	10 (77)	13 (77)	0.79
Regular alcohol consumption on admission, *n* (%)	10 (13)	4 (21)	2 (11)	0.66
Regular alcohol consumption at 12 months, *n* (%)	7 (44)	2 (15)	1 (14)	1.00
Diagnosis on admission				
STEMI/NSTEMI, *n* (%)	54/20 (72/27)	10/9 (53/47)	13/5 (68/26)	0.79 / 0.53
UAP, *n* (%)	1 (1)	0 (0)	1 (5)	1.00
STEMI onset-to-balloon time (min)	357 (226–803)	622 (240–872)	583 (224–1,373)	0.76
NSTE-ACS diagnosis to revascularization time				
<24 h	21	9	6	0.56
24–72 h	0	0	0	1.00
>72	0	0	0	1.00
Coronary angiography results				
Radial/femoral approach, *n* (%)	74/1 (99/1)	18/1 (95/5)	19/0 (100/0)	1.00 / 1.00
Mean number of significant coronary artery stenosis/patient, *n*	1.65 ± 0.76	1.58 ± 0.84	1.63 ± 0.83	0.72
Significant 1/2/3 vessel disease, *n* (%)	39/23/13 (52/31/17)	12/3/4 (63/16/21)	11/4/4 (58/21/21)	1.00 / 1.00 / 1.00
LM significant stenosis, *n* (%)	5 (7)	3 (16)	0 (0)	0.24
LAD significant stenosis, *n* (%)	47 (63)	14 (74)	14 (74)	1.00
RCA significant stenosis, *n* (%)	39 (52)	8 (42)	9 (47)	1.00
LCX significant stenosis, *n* (%)	29 (39)	6 (32)	5 (26)	1.00
Mean number of implanted DES/patient, *n*	1.51 ± 0.76	1.63 ± 0.68	1.67 ± 0.70	0.46
Mean diameter of implanted DES/patient, mm	3.05 ± 0.40	3.00 ± 0.36	3.04 ± 0.46	0.82
Mean total length of implanted DES/patient, mm	40.01 ± 25.48	38.42 ± 26.18	35.16 ± 16.51	0.89
Multivessel PCI, *n* (%)	8 (11)	2 (11)	2 (11)	1.00
Transthoracic echocardiogram				
Ejection fraction, %	50 (42–55)	52 (45–56)	46 (39–53)	0.07
Left ventricular end-diastolic/end-systolic diameter, mm	49.8 ± 5.6 /32.7 ± 5.4	51.3 ± 4.4 /32.2 ± 5.6	49.9 ± 5.5 /33.5 ± 5.5	0.20 0.84
Wall motion abnormality in two or more region/in one region at most, *n* (%)	35/40 (47/53)	4/15 (21/79)	8/11 (42/58)	0.35 / 0.61

Normally distributed parameters are given in a mean ± standard error of the mean (SEM) format, parameters with non-normal distributions were shown as median and interquartile ranges (IQR). Categorical values are given as *n* and percentage of the corresponding group (%). *p* = showed the statistical difference between the lower quartile and the upper quartile patient groups.

BMI, body mass index; BP, blood pressure; CAD, coronary artery disease; AMI, acute myocardial infarction; PCI, percutaneous coronary intervention; PAD, peripheral artery disease; STEMI, ST-segment elevation myocardial infarction; NSTEMI, non ST-segment elevation myocardial infarction; UAP, unstable angina pectoris; LM, left main coronary artery; LAD, left anterior descending artery; LCX, left circumflex artery; RCA, right coronary artery; DES, drug eluting stent; MACE, major adverse cardiovascular event; AE, adverse event; CV, cardiovascular; CABG, coronary artery bypass grafting; ER, emergency room; CCS, Canadian cardiovascular society angina grade; NYHA, New York Heart Association functional classification.

Our patients were predominantly male (84%) with 56.9 years of mean age, demonstrating that cardiovascular disease had an early onset in this population. Cardiovascular risk factors were broadly represented: baseline body mass index with median value of 27.5 kg/m^2^ reflecting an overweight population, 57% of the patients had hypertension, 48% had dyslipidaemia and 20% had diabetes mellitus. Additionally, 42% of the patients were current smokers and 61% of patients had a sedentary lifestyle. Analysis of ACS subtypes revealed a high-risk patient profile (54 ST-segment elevation myocardial infarction (STEMI), 20 non-ST-segment elevation myocardial infarction (NSTEMI) and 1 unstable angina) (Table 1). From indicative pain to revascularization (onset-to-balloon) time median value was 357 min for patients with STEMI. Due to all patients had high risk clinical status revascularization performed within 24 h in all NSTEMI and unstable angina cases.

All ACS cases were managed by urgent percutaneous coronary intervention (PCI) via transradial approach according to current revascularization guidelines. Although in 98.7% of the patients the culprit lesion was successfully treated with implantation of a second- or third generation drug eluting stent (DES), coronary angiography detected multivessel coronary disease in 48% of the cohort (Table 1).

After completing the CCR program, the cardiovascular risk status was efficiently improved; these modifications were persistent at the 12 months control (Table 1). The body mass index (BMI) decreased by 0.9 kg/m^2^. Half of the patients quit smoking completely and alcohol consumption was also decreased. Regular physical activity increased: 80% of the patients continued the 15–30 min/day physical training learned during the CCR program. These aforementioned parameters were improved by non-pharmacological methods like education, diet, stress management and exercise training during the CCR indicating the efficacy of the applied methods.

Similarly, pharmacological therapy showed good efficiency and adherence (see Table 2). High compliance with statin therapy was observed; 97% and 89% of the patients were taking statin at 3 and 12 months, respectively (Table 2). LDL cholesterol levels reached the actual targeted levels (Table 3; Supplementary Table S3). Concerning antiplatelet therapy, all patients were taking acetyl-salicylic acid during the entire follow-up (one patient had ASA allergy) and 87% of the patients were on P2Y_12_ receptor inhibitor therapy at 12 months. Drugs generally considered capable of preventing recurrent cardiovascular events, such as beta-blockers and ACE inhibitors/ARBs were extensively used in this population. Applied neurohormonal antagonist medications were similar between the two different subgroups over the study period (Supplementary Table S4).

**Table 2:** Antiplatelet/anticoagulant therapy throughout the study period and conventional medical therapy at enrolment in ACS patients with lower or upper quartile on-treatment epinephrine induced platelet aggregations.

Medical therapy				
	Total cohort (*n* = 75)	Epinephrine induced aggregations in the LQ (*n* = 19)	Epinephrine induced aggregations in the UQ (*n* = 19)	*p* =
Antiplatelet and anticoagulant therapy				
ASA, 100 mg				
At baseline, (%)	98	100	95	1.00
At 3 months, (%)	98	100	95	1.00
At 12months, (%)	98	100	95	1.00
Clopidogrel 75 mg/150 mg				
At baseline, (%)	78/7	85/10	80/10	1.00 / 1.00
At 3 months, (%)	80/0	90/0	84/0	1.00
At 12 months, (%)	75/0	92/0	76/0	0.81
Prasugrel, 10 mg				
At baseline, (%)	13	5	5	1.00
At 3 months, (%)	17	10	5	1.00
At 12 months, (%)	10	0	6	1.00
Ticagrelor, 90 mg				
At baseline, (%)	2	0	5	1.00
At 3 months, (%)	2	0	11	0.49
At 12 months, (%)	2	0	6	1.00
No P2Y_12_ inhibitor				
At baseline, (%)	0	0	0	1.00
At 3 months, (%)	1	0	0	1.00
At 12 months, (%)	13	8	12	1.00
VKA				
At baseline, (%)	11	11	11	1.00
At 3 months, (%)	11	5	5	1.00
At 12 months, (%)	11	5	5	1.00
Medical therapy at enrolment				
ACE-inhibitor/ARB, (%)	93/5	89/11	95/5	1.00/1.00
Beta–blocker, (%)	97	100	100	1.00
CCB, (%)	5	0	11	0.49
Nitrates, (%)	19	32	16	0.47
MRA, (%)	16	21	26	1.00
OAD/insulin, (%)	15/3	11/0	11/5	1.00 / 1.00
PPI, (%)	88	90	90	1.00
Diuretics, (%)	16	16	5	0.61
Statin,				
At baseline, (%)	97	95	95	1.00
At 3 months, (%)	97	95	100	1.00
At 12 months, (%)	89	85	88	1.00

Values are given as percentage of the corresponding total groups. *p* = showed the statistical difference between the lower quartile and the upper quartile patient groups.

ASA, acetylsalicylic acid; VKA, vitamin K antagonist; ACE, angiotensin converting enzyme; ARB, angiotensin receptor blocker; CCB, calcium channel blocker; MRA, mineralocorticoid receptor antagonist; OAD, oral antidiabetic therapy; PPI, proton-pump inhibitor.

**Table 3:** Laboratory parameters, cardiac and inflammatory biomarkers and salivary cortisol levels upon admission, at enrolment (96 h) and at 3- and 12-months follow-up in the total cohort and in the two patient groups with high or lower quartile epinephrine induced platelet aggregations.

Laboratory parameters				
	Total cohort	Epinephrine induced aggregations in the LQ	Epinephrine induced aggregations in the UQ	*p* =
Na (mmol/L)	142 (141–143)	141 (140–143)	142 (141–143)	0.51
K (mmol/L)	4.29 (4.06–4.52)	4.1 (4.0–4.37)	4.3 (4.1–4.6)	0.07
CN (mmol/L)	5.8 (5.1–6.6)	5.4 (4.5–6.1)	5.8 (5.0–6.7)	0.35
Creatinine (µmol/L)	87 (75–94)	91.0 (78.0–97.0)	87.0 (78.0–99.0)	0.84
Bilirubin (µmol/L)	10.35 (8.2–12.45)	9.3 (7.85–10.95)	**10.2** (**7.9–14.6)**	0.37
GGT (U/L)	29 (21–48.5)	30.5 (20.5–38.5)	**41.5** (**21.5–77.5)**	0.41
ALP (U/L)	80.5 (63–94)	86.5 (67.5–94.5)	84.0 (70.5–94.0)	0.83
TSH (µIU/ml)	1.43 (0.91–2.15)	0.95 (0.81–1.89)	**1.49** (**1.19–1.86)**	0.12
Red blood cell count (T/L)	4.66 (4.41–4.86)	4.72 (4.56–4.86)	4.65 (4.35–4.83)	0.37
Haemoglobin (g/L)	139 (130–147)	145 (136–149)	138 (133–145)	0.16
Haematocrit (L/L)	0.41 (0.39–0.43)	0.42 (0.41–0.43)	0.40 (0.39–0.42)	0.08
Platelet count (G/L)	211 (184–247)	198 (184–291)	216 (181–229)	0.60
Mean platelet volume (fl)				
On admission	10.5 (10.0–11.0)	10.4 (9.9–10.8)	10.7 (9.9–11.0)	0.52
At enrolment	10.7 (10.3–11.3)^a^	10.7 (10.3–11.2)	10.6 (10.3–11.3)^a^	0.77
At 3 months	10.8 (10.3–11.4)	10.4 (10.3–11.1)	10.6 (10.2–11.2)	0.88
At 12 months	10.5 (10–11.3)	10.5 (10.0–11.1)	10.6 (10.0–11.4)	0.43
Cardiac biomarkers				
hsTroponin T (ng/ml)				
On admission	384.8 (112.2–1,497)	199.4 (15.9–629.8)	**669.3** **(175–2,544)**^b^	0.07
At enrolment	963.5 (319.1–2,146)	831.1 (152.0–1,258.0)	**837.8** (**347.5–2,283.0)**	0.64
CK (U/L)				
On admission	304 (129–684)	201 (117–676)	380 (118–1,261)	0.44
At enrolment	149 (105–245)	148 (94–234)	170 (85–331)	0.78
At 3 months	122 (90–167)	134 (108–191)	132 (90–199)	0.98
At 12 months	118 (97–204)	107 (96–195)	121 (94–269)	0.85
CK-MB (U/L)				
On admission	11.9 (4.7–71.4)	6.2 (3.3–91.1)	**22.1** (**4.8–92.1)**	0.28
At enrolment	3.5 (2.2–5.7	3.11 (2.2–4.7)	2.9 (1.9–5.4)	0.78
ASAT (U/L)				
On admission	41 (28–69)	29 (20–58)	63 (30–92)***	0.02
At enrolment	29 (22–44)	22 (20–31)	**37** (**26–50)**	0.07
At 3 months	22 (18–27)	22 (18–28)	23 (18–27)	0.95
At 12 months	22 (18–27)	20 (19–30)	23 (18–25)	0.90
ALAT (U/L)				
On admission	30 (23–45)	23 (18–38)	43 (26–61)***	0.02
At enrolment	29 (21–42)	28 (18–33)	42 (22–62)***	0.04
At 3 months	27 (18–37)	29 (25–38)	26 (17–38)	0.57
At 12 months	25 (18–30)	24 (21–29)	29 (18–33)	0.80
LDH (U/L)				
On admission	383 (329–626)	350 (263–400)	466 (360–693)***	0.02
At enrolment	489 (380–708)	479 (345–558)	**493** (**404–775)**	0.21
At 3 months	197 (180–220)	186 (167–228)	**204** (**186–235)**	0.11
At 12 months	196 (174–211)	200 (165–208)	193 (176–206)	0.96
Inflammatory markers				
CRP (mg/L)				
On admission,	3.36 (1.06–10.8)	2.68 (0.32–11.56)	**4.67** (**2.74–23.3)**	0.10
At enrolment,	14.12 (5.39–34.4)^c^	15.27 (2.17–26.14)^c^	**12.30** (**5.02–50.49)**^c^	0.72
At 3 months,	1.76 (0.61–3.92)	1.54 (0.60–3.52)	1.52 (0.60–4.32)	0.89
At 12 months,	0.98 (0.6–2.67)	0.98 (0,60–1,08)	0.98 (0.62–3.43)	0.69
Leukocyte count (G/L)				
On admission,	9.8 (8.2–13.1)*	9.4 (8.2–11.4)*	10.8 (6.9–13.2)*	0.63
At enrolment,	8.0 (6.6–9.4)	8.2 (6.9–9.8)	7.6 (6.1–9.2)	0.21
At 3 months,	6.8 (5.7–8.7)	6.9 (5.8–8.1)	6.5 (5.7–8.9)	0.56
At 12 months,	6.7 (5.4–7.7)	6.7 (5.8–7.3)	6.37 (5.9–7.4)	0.71
Lipid parameters				
Total cholesterol (mmol/L)				
On admission,	5.0 (4.0–5.5)	5.0 (4.0–5.3)	5.0 (3.8–5.4)	0.94
At 3 months,	3.4 (3.0–4.1)^d^	3.5 (3.1–3.6)^d^	3.3 (2.9–4.1)^d^	0.87
At 12 months,	3.6 (3.0–4.4)^d^	**3.4** (**3.2–4.0)**	**4.0** (**3.1–5.0)**	0.43
LDL-cholesterol (mmol/L)				
on admission,	3.0 (2.4–3.8)	2.9 (2.4- 3.1)	3.2 (1.8–4.1)	0.79
at 3 months,	1.7 (1.4–2.4)^d^	1.7 (1.5–2.2)^d^	1.7 (1.2–2.5)^d^	0.77
at 12 months,	1.8 (1.4–2.5)^d^	1.8 (1.4–2.1)^d^	2.1 (1.4–2.6)^d^	0.43
HDL-cholesterol (mmol/l)				
On admission,	1.1 (1.0–1.3)	1.2 (1.0–1.3)	1.1 (1.1–1.3)	0.94
At 3 months,	1.0 (0.9–1.2)^d^	1.0 (0.8–1.2)^d^	1.0 (0.9–1.2)	0.95
At 12 months,	1.1 (1.0–1.3)	1.2 (1.0–1.3)	1.1 (0.9–1.2)	0.20
Triglyceride (mmol/L)				
On admission,	1.5 (1.2–1.9)	1.8 (1.2–1.9)	1.6 (1.2–2.5)	0.62
At 3 months,	1.2 (1.0–1.7)^d^	1.2 (1.0–1.6)	1.3 (1.0–2.0)^d^	0.64
At 12 months,	1.2 (0.9–2.2)	1.3 (1.0–1.4)	1.6 (1.0–2.4)	0.14
Salivary cortisol levels				
At enrolment,	11.0 (8.0–14.2)	9.4 (7.8–12.2)	**11.5** (**8.9–13.5)**	**0**.**37**
At 3 months,	11.3 (7.65–14.6)	10.5 (9.0–13.3)	**12.1** (**7.8–14.9)**	**0**.**84**
At 12 months,	7.8 (5.45–12.85)**	6.8 (4.45–13.5)	**8.5** (**5.4–13.7)**	**0**.**27**

Values are medians ± lower and upper quartile ranges. *p* = showed the statistical difference between the lower quartile and the upper quartile patient groups. Numbers in bold indicate that the parameters are higher in the upper quartile group, but the difference did not reach the statistical significance (Mann–Whitney tests, *n* = 19).

Na, sodium; K, potassium; CN, carbamide; GGT, gamma-glutamyl transferase; ALP, alkaline phosphatase; TSH, thyroid-stimulating hormone; CK, creatin kinase; CK–MB, creatin kinase muscle/brain isoform; ASAT, aspartate aminotransferase; ALAT, alanine aminotransferase; LDH, lactat dehydrogenase; CRP, C-reactive protein; HDL, high-density lipoprotein; LDL, low-density lipoprotein.

* Leukocyte count was significantly higher (*p* < 0.05) on admission compared to enrolment or 3 and 12 months controls.

** Indicate that salivary cortisol levels at 12 months are significantly reduced compared to baseline and to 3 months follow-up, *p* < 0.05.

*** *p* < 0.05 ASAT, ALAT and LDH levels are higher in the upper quartile group.

^a^ MPV values are higher at enrolment than on admission, *p* < 0.05, for the LQ group *p* = 0.07.

^b^ *p* = 0.07, *n* = 19, Mann–Whitney test between troponin levels on admission in lower and upper quartile patients.

^c^ CRP levels are significantly lower on admission than at enrolment with *p* values are <0.05 in the total cohort and the lower quartile patient group. *p* = 0.058 in the upper quartile patients (Wilcoxon matched pairs test).

^d^ *p* < 0.05 Wilcoxon matched pairs test between on admission and 3 or 12 months values,

Despite the initially high cardiovascular risk profile, the major cardiovascular event rate remained low at one year follow-up and all patients were in NYHA I or II class at 12 months (Supplementary Table S1).

### 3.2 Kinetics of platelet activation and salivary cortisol levels after ACS

**Figure 1:**
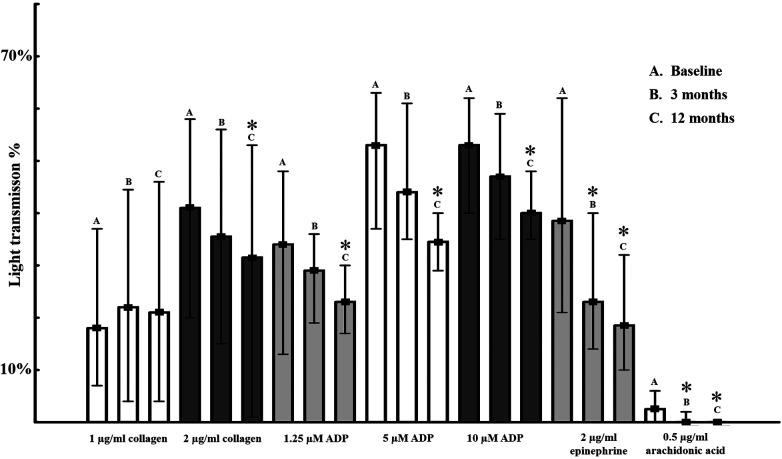
**Platelet aggregations induced by different platelet agonists in acute coronary syndrome patients at enrolment, 3 months and 12 months**. Platelet aggregation determined by light transmission aggregometry in platelet rich plasma by different concentrations of platelet agonists. Median values in percentage and interquartile ranges are shown as columns and lines respectively *<0.05 compared to baseline value.

Platelet aggregations induced by different platelet agonists at baseline, 3 and 12 months are shown in Figure 1. General platelet functionality was determined using several agonists at different concentrations (collagen 1 and 2 μg/ml, ADP 1.25, 5, 10 μM, epinephrine 2 μg/ml and arachidonic acid at 0.5 μg/ml concentrations).

Baseline values—determined at enrolment, 84–96 h after the acute atherothrombotic event—clearly indicate platelet hyperactivity, accompanying acute coronary syndromes despite the applied dual antiplatelet therapy (Table 2). Interestingly, initial high platelet activity showed a uniformly, slowly decreasing pattern over time in spite of effective constant P2Y_12_ receptor/COX-1 inhibition (Figure 1). At 3 months only 2 μg/ml epinephrine and 0.5 μg/ml arachidonic acid showed significant decrease in platelet reactivity (*p* < 0.05). Overall, gradually decreasing platelet function reached statistical significance at 12 months: aggregations induced by 2 μg/ml collagen, 1.25, 5, 10 μM ADP, 2 μg/ml epinephrine and 0.5 μg/ml arachidonic acid were lower compared to baseline value, *p* < 0.05. General loss of platelet hyperactivity is not linked to modifications of antiplatelet therapy, as shown in Table 2.

Although all platelet agonists showed reduced response at 12 months, the phenomenon was more rapid (already significant at 3 months) and more pronounced in case of epinephrine compared to collagen or ADP (Figure 1). Moreover, baseline epinephrine-induced aggregations showed significant correlation with most of the agonists at baseline and at 3 months. The correlation was mostly lost at 12 months, suggesting a diminishing impact of the sympathetic neurosystem over time (Table 4).

**Table 4:** Correlation between aggregations induced by baseline 2 μg/ml epinephrine and different agonists at baseline, 3 months, and 12 months.

Agonist	Correlation with epinephrine induced platelet aggregation at baseline
Baseline	
1 μg/ml collagen	0.364*
2 μg/ml collagen	0.540*
1.25 μM APD	0.190
5 μM ADP	0.330*
10 μM ADP	0.339*
2 μg/ml epinephrine	1.00
0.5 μg/ml arachidonic acid	−0.018
3 months	
1 μg/ml collagen	0.407*
2 μg/ml collagen	0.229*
1.25 μM APD	0.219
5 μM ADP	0.155
10 μM ADP	0.258*
2 μg/ml epinephrine	0.332*
0.5 μg/ml arachidonic acid	0.306*
12 months	
1 μg/ml collagen	0.201
2 μg/ml collagen	0.286*
1.25 μM APD	0.089
5 μM ADP	0.162
10 μM ADP	0.163
2 μg/ml epinephrine	0.364*
0.5 μg/ml arachidonic acid	0.104

* Marked correlation are significant at *p* < 0.05.

It is important to note, that our patient population consisted of STEMI and high-risk NSTEMI patients (Table 1). In respect to baseline platelet aggregation values, we found no difference between these two patient groups (Supplementary Table S5).

In our study, salivary cortisol level—as the final effector of the stress-mediated hypothalamic–pituitary–adrenal axis (HPA)—was also determined. Similarly, to platelet aggregations, cortisol levels were the highest at enrolment and showed slow decrease over time being statistically significant at 12 months despite the relatively small sample size (*p* < 0.05, Table 3).

### 3.3 Patient characteristics and laboratory parameters within subgroups with epinephrine—induced platelet aggregations in the lower- and upper quartile

Epinephrine-induced aggregations showed rapid and pronounced decrease after the acute cardiovascular event. To identify subjects with high or low adrenergic activity and detect individual characteristics, study patients were classified into quartiles based on epinephrine induced aggregations at enrolment. Patient subgroups with epinephrine induced aggregation in the lower quartile (EIA—LQ) and in the upper quartile (EIA—UQ) were compared in different settings (Tables 1, 3).

No statistical difference was found between EIA—LQ and EIA—UQ patients concerning gender, age, coronary angiography results and PCI methods (Table 1). There was numerically more diabetes mellitus, hyperlipidaemia, and higher diastolic blood pressure in EIA—UQ, indicating a higher risk population (Table 1). Medical therapy did not significantly differ between the two groups; however, more effective P2Y_12_ inhibitors were frequently used in patients in the upper quartile (Table 2).

Concerning laboratory results during the acute cardiovascular event, cardiac biomarkers/necroenzymes such as high sensitivity Troponin T (*p* = 0.07), creatine kinase (CK), creatine kinase muscle/brain (CK-MB) isoform, aspartate aminotransferase (ASAT, *p* < 0.05) and lactate dehydrogenase (LDH, *p* < 0.05) were higher on admission in the EIA—UQ group (Table 3). Similarly, general inflammatory markers accompanying ACS, like high sensitivity C-reactive protein (CRP) and leukocyte numbers were also elevated in the EIA—UQ group (Table 3). Moreover, in upper quartile patients the baseline left ventricular ejection fraction (LVEF) was lower (*p* = 0.07) and wall motion abnormalities (WMA) were more extended compared to EIA—LQ group (Table 1). In addition, there was a trend towards more adverse cardiac events in the EIA—UQ subgroup (Supplementary Table S1). These findings might indicate that patients with increased platelet responsiveness to adrenergic activators have more extended cardiac necrosis on admission, with elevated cardiac necroenzymes and inflammatory markers. It is tempting to speculate, that lower ejection fraction, more pronounced wall motion abnormalities and more frequent cardiac events were observed as a consequence in these individuals (Table 1).

Finally, median salivary cortisol level, used as marker of the stress-mediated HPA axis, was also higher in the EIA—UQ patient group at enrolment and at 3 and 12 months (Table 3). Although the difference did not reach the statistical significance (case number in the quartile subgroups was 19), this observation is in line with the concept of simultaneously increased stress hormones/adrenergic platelet activity in acute coronary patients.

In summary, results suggest that high adrenergic platelet activity is accompanied with distinct cardiac necroenzymes, inflammatory marker and clinical parameter profile in acute coronary syndrome patients.

### 3.4 Platelet aggregations in patient groups with high- or low epinephrine-induced platelet aggregation

**Figure 2:**
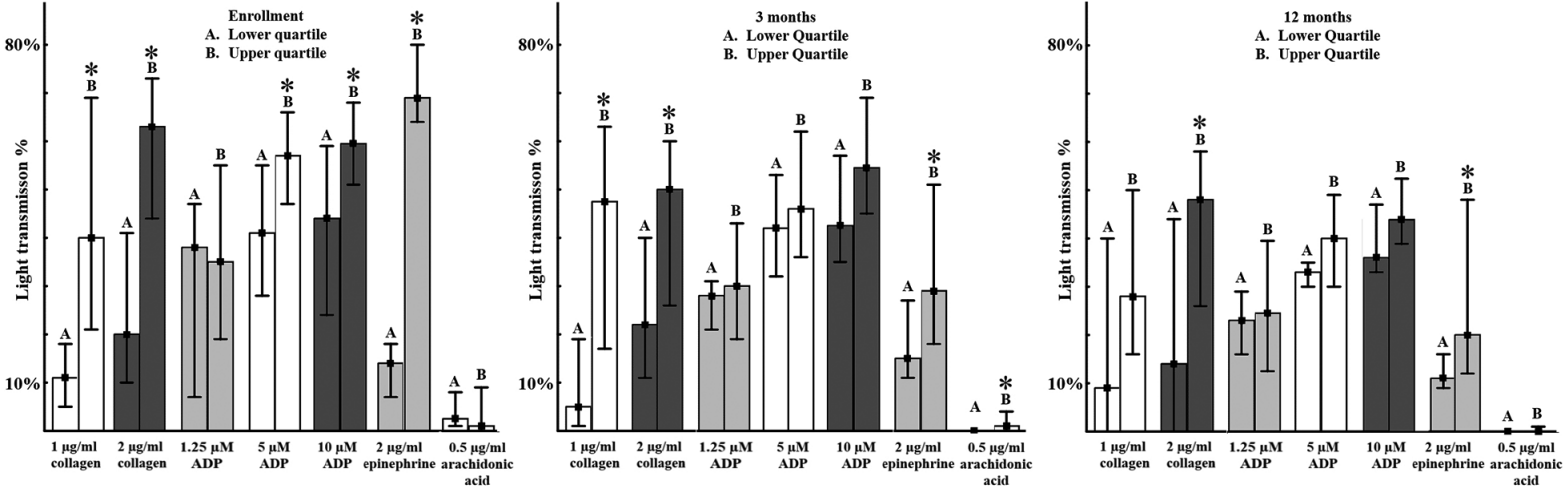
**Platelet aggregations induced by different platelet agonists according to the baseline epinephrine induced aggregation quartile at enrolment, 3 months and 12 months**. Platelet aggregation determined by light transmission aggregometry in platelet rich plasma by different concentrations of platelet agonists. Median values in percentage and interquartile ranges are shown as columns and lines respectively *<0.05 compared to baseline value.

Platelet aggregations induced by different platelet agonists in the EIA lower- or upper quartile patient subgroups are shown in Figure 2. The first panel demonstrates that at enrolment, particularly ADP and collagen induced aggregations were significantly higher in the EIA—UQ than in the EIA—LQ group, indicating that high adrenergic activation is accompanied by general increased platelet reactivity to other agonists.

Importantly, platelet aggregations in these two subgroups showed the same pattern at 3- and 12-months measurements as well (Figure 2). Even though patients were characterized to subgroups based on their initial adrenergic platelet response at enrolment, adrenalin-induced aggregations in individuals in the upper patient quartile remained significantly higher throughout the 12 months study period (*p* < 0.05). Moreover, general increased platelet reactivity in response to different concentrations of ADP and collagen also persisted at 3- and 12-months follow-up in the EIA—UQ group, compared to the lower quartile (*p* < 0.05, Figure 2).

These results indicate that increased epinephrine-induced aggregations efficiently identify individuals with general platelet hyperreactivity during the acute phase and more importantly, might predict sustained higher general platelet reactivity up to one-year follow-up.

### 3.5 Effect of selective adrenergic inhibition

**Figure 3:**
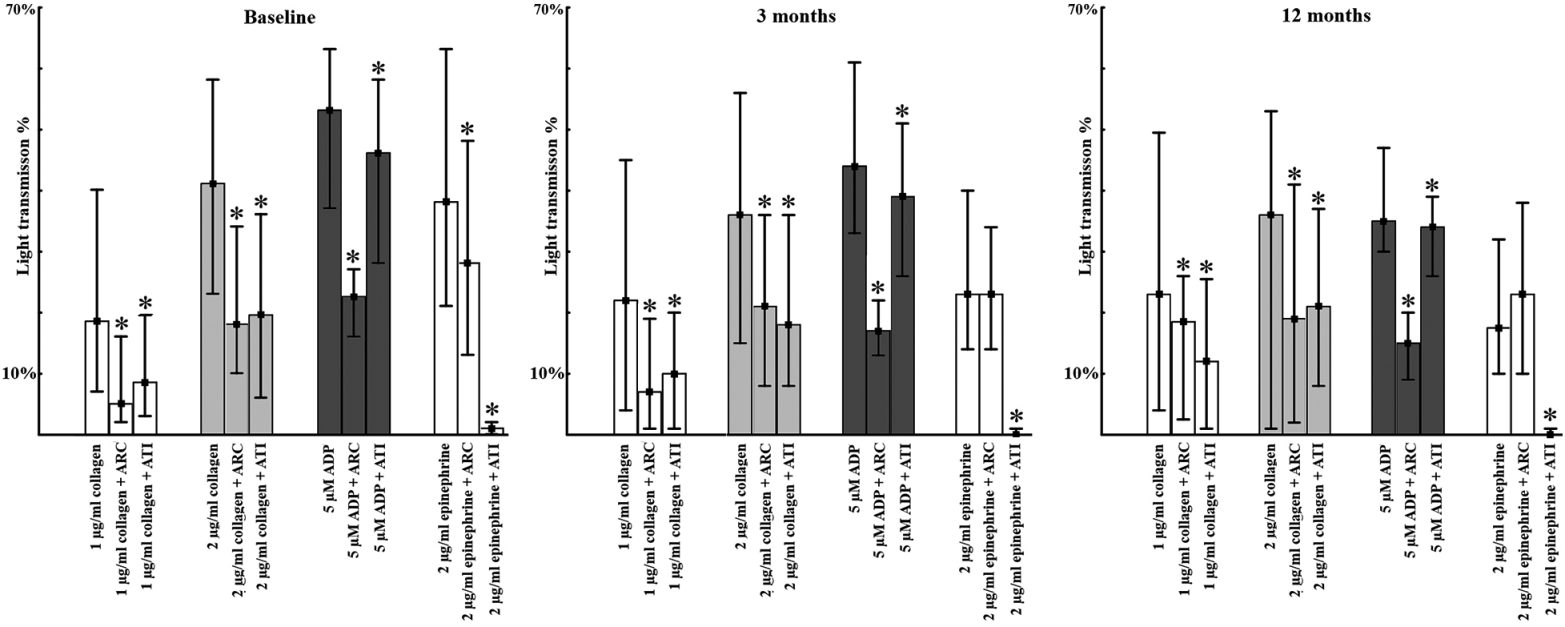
**Platelet aggregations induced by different platelet agonists and antagonist in acute coronary syndrome patients at enrolment, 3 months and 12 months**. Platelet aggregation determined by light transmission aggregometry in platelet rich plasma by different concentrations of platelet agonists. Median values in percentage and interquartile ranges are shown as columns and lines respectively *<0.05 compared to baseline value.

Atipemazole, a selective inhibitor of the alpha-2 adrenergic receptor fully inhibited epinephrine induced platelet aggregations in citrated plasma *in vitro* at baseline and at follow-ups, as expected (*p* < 0.05, Figure 3). Interestingly, it also significantly reduced platelet activity induced by other agonists as well—collagen and ADP-induced platelet aggregation were significantly lower in the presence of atipemazole at baseline and at 3- and 12-months follow-up (*p* < 0.05), despite of the concomitant oral P2Y_12_ receptor antagonist medical therapy (Figure 3). Furthermore, the effect of atipemazole was most pronounced on 1 ug/ml, 2 ug/ml collagen-induced aggregations, where its efficacy was comparable to the inhibitory capacity of cangrelor, a selective P2Y_12_ inhibitor used *in vitro* as well (Figure 3). In contrast, atipemazole showed significant, but less effective inhibition on ADP-induced aggregations, where cangrelor was more potent, as expected. Remarkably, cangrelor inhibited epinephrine-induced aggregations only at the acute phase; the effect was diminished at the 3- and 12-months controls (Figure 3).

## 4 Discussion

After acute coronary syndromes current guidelines highly recommend a multidisciplinary approach to reduce residual modifiable cardiovascular risk factors and to decrease cardiovascular morbidity and mortality (3, 26). Complex cardiac rehabilitation (CCR) after an ACS episode is capable to offer comprehensive and effective amendment of the traditional risk factors (4, 27). Our CCR program focused on diet, exercise training, education and stress management and as a result, pronounced reduction of conventional cardiovascular risk factors was detected. BMI was decreased, half of the patients quit smoking and performed exercise training regularly in short- and mid-term (3 and 12 months).

While several studies revealed that patients often discontinue their medication shortly after ACS (28, 29), high adherence to medical therapy was detected within the CCR program in our study. Essential drugs such as statins, ACEI/ARBs and beta-blockers were applied, and patients reached the targeted systolic and diastolic blood pressure and recommended LDL-C levels. Dual antiplatelet therapy was maintained as well. These pharmacological and non-pharmacological therapies went hand in hand and resulted in an excellent functional NYHA classification status and low cardiovascular event rates, confirming the benefits of early rehabilitation.

Acute coronary syndrome represents an acute atherothrombotic event characterized by increased platelet activation leading to the formation of intracoronary thrombi (30). Optimal level of platelet inhibition is an essential element of the medical therapy, especially if intracoronary stents are implanted. Current ACS guidelines recommend 12 months of dual antiplatelet therapy (DAPT) to reduce the risk of further adverse events (31). Our study demonstrated high general platelet reactivity four days after ACS and percutaneous coronary intervention, decreasing gradually and slowly over time, up to one year. This observation supports the recommended period of DAPT and might be a result of multiple factors, such as cessation of the acute atherothrombotic event and lifestyle changes (4).

On-treatment platelet function varies from individual to individual and generally considered to be an independent risk factor of recurrent ischemic activity (6, 32).

Although all platelet aggregometry measurements were performed uniformly on patients on dual antiplatelet therapy, individual differences were considerable in our study as well. Particularly epinephrine-induced aggregations showed wide variability especially in the acute phase of coronary syndromes. Moreover, these aggregations were proper indicators of general platelet hyperactivity: patients with high adrenergic activity had high response to other platelet agonists (collagen, ADP). This phenomenon was observed in the acute phase, but more importantly, patients with high initial platelet activity at enrolment had significantly higher remaining platelet reactivity at 3 months and at 12 months as well—despite the above described, uniformly applied pharmacological and non-pharmacological secondary prevention methods. This observation indicates that platelet activity is determined by individual internal factors, evolving their effect in short- and long-term, emphasizing the significance of adrenergic response during the acute and chronic phase.

While the role of alpha-adrenergic activation in general platelet activity has been suggested previously, detailed data in ACS patients were lacking. Previous study showed that despite DAPT *in vitro*, combination of low-dose epinephrine significantly potentiates, while selective alpha-2 receptor blockade by atipemazol significantly inhibits ADP- and collagen induced aggregations in chronic coronary syndrome (16). Based on our data concerning the specific alpha-2 adrenergic receptor inhibitor atipemazole, we concluded that potent adrenergic inhibitory effect can be observed through the entire study period, with the highest effectivity of alpha-2 inhibition on collagen-induced aggregations in ACS population as well. As demonstrated by *in vitro* use of cangrelor (specific *in vitro* direct P2Y_12_ inhibitor on addition to oral DAPT), the inhibitory effect of alpha-2 receptor blockade was comparable with the efficacy of full P2Y_12_ inhibition—indicating that collagen-induced aggregations rely significantly on other amplification pathways. Although they are not able to induce full platelet aggregation in washed platelets, catecholamines were shown to influence the platelet cAMP content via Gi protein activation and strongly potentiate aggregations induced by other agonists (33, 34). Since collagen fibers are the most abundant type of fibers in the extracellular matrix of vascular connective tissue, it is tempting to speculate that actual catecholamine concentrations within the vascular microenvironment might influence the thrombogenic capacity of the subendothelial matrix during acute plaque rupture. This effect might be rapid, transient, and adaptable according to the actual general stress level, since catecholamine clearance from the circulation is a fast, well-regulated process (13, 35).

Rapid activation of the adrenergic receptor on the platelet surface might contribute to the development of platelet hyperactivity in short and long-term as well. Over the long term, high adrenergic activity might maintain general platelet hyperreactivity, thereby contributing to recurrent ischaemic cardiac events (11–13). Initial epinephrine-induced aggregations significantly correlated with aggregations induced by other agonists at later time-points as well. These observations indicate that catecholamines modifying effect is most pronounced in the acute phase but can be detected up to one year. During the acute phase, patients with ACS require immediate hospitalization and intervention—further increasing the level of perceived stress and consecutive hormone secretion, resulting in a vicious circle. This concept is supported by the gradual decline in salivary cortisol levels, measured at enrolment and at 3- and 12-months controls in our study as well.

Humans respond to environmental perturbations with a stress signal that facilitates physiological adaptation to the stressor and maintains homeostasis. A major component of the homeostatic reaction involves the simultaneous activation of the sympathetic nervous system and the hypothalamic-pituitary-adrenal (HPA) axis, an intricate neuroendocrine mechanism regulating numerous physiological processes (18).

The HPA axis consists of a cascade of endocrine pathways that respond to specific negative feedback loops involving the hypothalamus, anterior pituitary gland, and adrenal gland (12). Objective measurement of the individual's stress level is challenging—however, biological parameters might help to detect activation of the hypophysis-adrenal gland system. Cortisol is the final product of stress mediated HPA axis activation, can be measured in blood, hair, urine, or saliva. Salivary cortisol converted to cortisone, provides a stable form that more reliably estimates serum cortisol levels. (36). Both serum and salivary cortisol levels showed correlation with the impact of externally induced stress (37) and previous works shown that serum cortisol elevation is related to the severity of myocardial infarction (20, 21, 38). In our study, elevated salivary cortisol levels were detected at enrolment, indicating that ACS is a significant stressor, capable of inducing concomitant activation of both the HPA axis and catecholamine-related platelet aggregability.

Based on our data, epinephrine-induced aggregations are hugely variable in the acute phase. Despite uniformly applied antiplatelet medical therapy, patients in the higher quartile (EIA—UQ group) demonstrated more than fourfold higher median epinephrine induced aggregations than those in the lower quartile. Interestingly, the variability decreased with time in the chronic phase, indicating that acute stress has the most potent effect, creating considerable individual heterogeneity. Consistent with our results, previous studies showed that both acute and chronic stress represents an independent cardiovascular risk factor and stress management strategies can help to mitigate its effect (19, 20, 22, 23). Clinical characteristics of the EIA—UQ patient subgroup indicated accentuated initial cardiovascular risk: besides high incidence of hypertension, dyslipidaemia and diabetes mellitus, baseline cardiac biomarker (hsTnT) and necroenzymes levels (CK, CKMB, ASAT, LDH) were also elevated. Extensive myocardial necrosis caused by occlusion of a dominant epicardial coronary artery, or a longer coronary reperfusion delay time has led to reduced left ventricular ejection fraction and more extent wall motion abnormalities, as detected in this patient group. In addition, in EIA—UQ group hsCRP levels and leukocyte count were also higher, in line with the well-known triggering/consecutive role of inflammation in acute atherothrombotic pathways (39). Importantly, although not reaching statistical difference due to the low case number, serum cortisol levels were elevated in the EIA-UQ group, suggesting co-activation of both major stress-mediated pathways, namely the HPA axis and the sympathetic nervous system.

Extent of the myocardial necrosis and high residual platelet reactivity are both associated with poor short and long-term prognosis in the ACS population (6, 40). Although our study was not powered to determine clinical outcome and cardiovascular mortality differences between the patient subgroups, we observed more cardiac event in the EIA—UQ group. The aforementioned characteristics of patients with high adrenergic platelet reactivity, such as strengthened acute or chronic stress response, more comorbidities, extensive myocardial necrosis, higher inflammatory response and higher general platelet reactivity are circularly deteriorating factors and as a result, might convey inferior cardiovascular prognosis in this population. Previously, clinical studies focused on measurement of the P2Y_12_ ADP receptor inhibitors efficacy. With the availability of more potent and more homogenously acting new P2Y_12_ receptor inhibitory drugs, measurement of ADP-induced aggregations/activation might provide less information concerning general residual platelet reactivity. It is tempting to hypothesize, that increased initial epinephrine—induced aggregations might indicate long-term cardiovascular risk and might help to identify the most vulnerable patient population. To establish this new parameter, future larger scale studies—powered to properly detect cardiovascular mortality, survival or adverse cardiac events in this population—are needed.

Currently, due to abundant presence of alpha-2 adrenergic receptors in numerous cell types, selective inhibition of platelet adrenergic function is an unmet need in pharmacological therapy and could be a new target for drug development. Until then, only non-pharmacological strategies are available to reduce activation of the sympathetic neuroendocrine system in response to ACS-related stress. Although in our study all patients underwent early, complex cardiac rehabilitation with uniformly applied stress management techniques, stress-related parameters decreased significantly only after 3 months. Future studies might determine the optimal method/timing of these techniques during cardiac rehabilitation and most importantly, whether cardiovascular mortality can be reduced after ACS by these non-pharmacological interventions.

Our study has limitations; first of all, patient enrolment process inevitably led to selection bias. We collected patients with “good health behaviour”, since all study individual agreed to participate in a more sophisticated CCR program and several control visits. Considered as the major limitation factor due to financial considerations, is our small sample size (concerning especially salivary cortisol levels), detecting differences between patient subgroups as trends, especially when certain biological parameters are known to have large variability. For the same reason we cannot directly demonstrate a correlation between platelet function in adrenergic agonist-induced subgroup analysis and salivary cortisol levels. Since several factors (e.g., circadian rhythm, sampling time, stress related to sample administration) might influence it, collection of salivary cortisol—despite our efforts—is challenging to standardise. In addition, the study followed European and American cardiological guidelines, however, it is important to note that due to national financing regulations, patients treated with potent P2Y_12_ inhibitors (ticagrelor or prasugrel) were underrepresented. Finally, we enrolled both STEMI and high-risk NSTEMI patients, since medical therapy—including early invasive approach in the case of NSTEMI patients—was homogenous in this population. Subgroup analysis revealed no difference between these patient groups in respect to baseline platelet aggregometry values, indicating that the presence of ST elevation as a single factor is not able to determine platelet reactivity. However, in the future independent analysis of larger patient populations might be needed to establish platelet adrenergic activity as a parameter conveying cardiovascular risk in the full spectrum of acute coronary syndromes.

In summary, initial, slowly diminishing, systemic, parallel activation of the HPA axis and the sympathetic neuroendocrine system was detected in acute coronary syndrome patients, with considerable individual heterogeneity. Based on elevated initial epinephrine-induced aggregations we defined an acute coronary patient group with more pre-existing comorbidities, extensive myocardial necrosis, higher inflammatory parameters, elevated salivary cortisol levels and higher initial and residual long-term general platelet reactivity. The selective alpha-2 adrenergic inhibitor *in vitro* reduced platelet aggregation induced by other platelet agonists as well—supporting the assumption that catecholamines might serve as prompt platelet function modulators and high adrenergic platelet activity is related or might be a marker of elevated cardiovascular risk. Therefore, we emphasize the importance of effective stress management techniques offering a non-pharmacological pathway to optimize platelet function during secondary prevention in these patients. Larger scale studies are needed to confirm the clinical relevance and impact on cardiovascular mortality of catecholamine-related platelet activation pathways in these human settings.

## Data Availability

The raw data supporting the conclusions of this article will be made available by the authors, without undue reservation.
