# Supplementary material for: High platelet adrenergic activity and concomitant activation of the pituitary/medullar axis as alarming laboratory parameters in ACS survivors—the STRESS-AMI study
Source: Front Cardiovasc Med. 2024 Feb 21;11:1338066. doi: 10.3389/fcvm.2024.1338066 (PMC10914969; doi:10.3389/fcvm.2024.1338066)
Supplement: Supplementary file 1 [file Table1.docx]

**Supplementary Table 1.**

| **Demographic data** | **Total**  **cohort**  **(n=75)** | **LQ (n=19)** | **Q2 (n=19)** | **Q3 (n=18)** | **UQ (n=19)** |
| --- | --- | --- | --- | --- | --- |
| Male/female, n (%) | 63/12 (84/16) | 17/2 (89/11) | 17/2 (89/11) | 12/6 (67/33) | 17/2 (89/11) |
| Age, years | 56.9 ± 8.8 | 55.1 ± 6.4 | 57.9 ± 9.7 | 59.7 ± 9.8 | 55.9 ± 10.1 |
| BMI on admission, (kg/m^2^) | 27.5  (25.2-32.1) | 27.1  (25.3-33.3) | 27.6  (24.5-30.0) | 27.5  (25.9-32.0) | 27.7  (24.9-33.8) |
| BMI at 12 months, (kg/m^2^) | 26.6  (24.1-30.4) | 25.8  (24.6-30.0) | 27.6  (23.0-30.4) | 25.3  (22.0-28.7) | 28.0  (24.2-37.0) |
| Hypertension, n (%) | 43 (57) | 11 (58) | 10 (52) | 11 (61) | 11 (58) |
| Systolic BP on admission, (Hgmm) | 135.9 ± 19.9 | 138.3 ± 23.4 | 130.7 ± 16.7 | 135.7 ± 23.9 | 138.8 ± 14.7 |
| Diastolic BP on admission, (Hgmm) | 85.3 ± 12.9 | 80.6 ± 15.0 | 82.6 ± 9.9 | 87.3 ± 15.3 | 85.8 ± 11.2 |
| Diabetes mellitus, n (%) | 15 (20) | 2 (11) | 2 (11) | 6 (33) | 5 (26) |
| Hyperlipidemia, n (%) | 36 (48) | 8 (42) | 7 (37) | 9 (50) | 12 (63) |
| Hyperuricemia, n (%) | 9 (12) | 3 (16) | 1 (5) | 3 (17) | 2 (11) |
| Previous CAD, n (%) | 21 (28) | 5 (26) | 5 (26) | 7 (39) | 4 (21) |
| Previous AMI/PCI, n (%) | 6/10 (8/13) | 2/3 (11/16) | 3/3 (16/16) | 1/3 (6/17) | 0/1 (0/5) |
| Previous stroke/TIA, n (%) | 2/3 (3/4) | 0/1 (0/5) | 1/1 (5/5) | 0/1 (0/6) | 1/0 (5/0) |
| PAD, n (%) | 2 (3) | 1 (5) | 0 (0) | 0 (0) | 1 (5) |
| Thyroid disease, n (%) | 8 (11) | 3 (16) | 3 (16) | 1 (6) | 1 (5) |
| Smoking previously, n (%) | 17 (22) | 3 (16) | 3 (16) | 5 (28) | 6 (32) |
| Current smoker, n (%) | 32 (42) | 13 (68) | 5 (26) | 8 (44) | 6 (32) |
| Smoking at 12 months, n (%) | 17 (25) | 7 (44) | 3 (16) | 4 (22) | 3 (16) |
| Regular exercise on admission, n (%) | 29 (39) | 8 (42) | 6 (32) | 6 (33) | 9 (47) |
| Regular exercise at 12 months, n (%) | 44 (80) | 10 (77) | 12 (63) | 9 (50) | 13 (77) |
| Regular alcohol consumption on admission, n (%) | 10 (13) | 4 (21) | 3 (16) | 1 (6) | 2 (11) |
| Regular alcohol consumption at 12 months, n (%) | 7 (44) | 2 (15) | 2 (11) | 2 (11) | 1 (14) |
| **Diagnosis on admission** |  |  |  |  |  |
| STEMI/NSTEMI, n (%) | 54/20 (72/27) | 10/9 (53/47) | 15/4 (79/21) | 16/2 (89/11) | 13/5 (68/26) |
| UAP, n (%)  STEMI Onset-to-balloon time (min)  NSTE-ACS diagnosis to revascularization time  <24h  24-72h  >72 | 1 (1)  357 (226-803)  21  0  0 | 0 (0)  622 (240-872)  9  0  0 | 0 (0)  242 (124-560)  4  0  0 | 0 (0)  373 (233-632)  2  0  0 | 1 (5)  583 (224-1373)  6  0  0 |
| **Coronary angiography results** | | | | | |
| Radial/femoral approach, n (%) | 74/1 (99/1) | 18/1 (95/5) | 19/0 (100/0) | 18/0 (100) | 19/0 (100/0) |
| Mean number of significant coronary artery stenosis/patient, n | 1.65 ± 0.76 | 1.58 ± 0.84 | 1.73 ± 0.56 | 1.67 ± 0.84 | 1.63 ± 0.83 |
| Significant 1/2/3 vessel disease, n  (%) | 39/23/13  (52/31/17) | 12/3/4  (63/16/21) | 6/12/1  (32/63/5) | 10/4/4  (56/22/22) | 11/4/4  (58/21/21) |
| LM significant stenosis, n (%) | 5 (7) | 3 (16) | 1 (5) | 1 (6) | 0 (0) |
| LAD significant stenosis, n (%) | 47 (63) | 14 (74) | 10 (56) | 9 (50) | 14 (74) |
| RCA significant stenosis, n (%) | 39 (52) | 8 (42) | 11 (58) | 11 (61) | 9 (47) |
| LCX significant stenosis, n (%) | 29 (39) | 6 (32) | 10 (56) | 8 (44) | 5 (26) |
| Mean number of implanted DES/patient, n | 1.51 ± 0.76 | 1.63 ± 0.68 | 1.38 ± 0.83 | 1.56 ± 0.85 | 1.67 ± 0.70 |
| Mean diameter of implanted DES/patient, mm | 3.05 ± 0.40 | 3.00 ± 0.36 | 3.07 ± 0.31 | 3.05 ± 0.33 | 3.04 ± 0.46 |
| Mean total length of implanted DES/patient, mm | 40.01 ± 25.48 | 38.42 ± 26.18 | 41 ± 24.5 | 45.1 ± 33.3 | 35.16 ± 16.51 |
| Multivessel PCI, n (%) | 8 (11) | 2 (11) | 2 (11) | 2 (11) | 2 (11) |
| **Transthoracic echocardiogram** |  |  |  |  |  |
| Ejection fraction, % | 50 (42-55) | 52 (45-56) | 54 (32-59) | 46 (39-52) | 46 (39-53) |
| Left ventricular end-diastolic / end-systolic diameter, mm | 49.8 ± 5.6 /  32.7 ± 5.4 | 51.3 ± 4.4 /  32.2 ± 5.6 | 50.0 ± 5.8 /  33.8 ± 5.8 | 48.3 ± 6.5 /  30.1 ± 4.0 | 49.9 ± 5.5 /  33.5 ± 5.5 |
| Wall motion abnormality in two or more region / in one region at most, n (%) | 35/40  (47/53) | 4/15  (21/79) | 8 /11  (42/58) | 15/3  (83/17) | 8/11  (42/58) |
| **MACE/AE at 12 months** |  |  |  |  |  |
| Recurrent AMI/Stroke/CV Death, n | 0/0/2 | 0/0/0 | 0/0/1 | 0/0/1 | 0/0/0 |
| Recoronarography elective/urgent, n | 4/1 | 0/0 | 1/0 | 1/0 | 2/1 |
| Coronary artery restenosis, n | 4 | 0 | 1/0 | 1/0 | 2 |
| Revascularization:  Elective PCI/Acute PCI/CABG, n | 3/1/3 | 0/0/1 | 1/1 | 1/1/1 | 1/1/1 |
| Rehospitalization for chest pain, n | 1 | 0 | 0 | 0 | 1 |
| ER visit due to chest pain/dyspnoe, n | 1/1 | 0/0 | 0/0 | 0/1 | 1/0 |
| CCS classification I/II, n | 52/3 | 12/1 | 13/0 | 10/2 | 17/0 |
| NYHA I/II, at 12 months, n | 50/5 | 13/0 | 12/1 | 9/3 | 16/1 |

**Supplementary Table 1. Clinical data and demographic parameters of the complete patient population and the four subgroups according to baseline epinephrine induced platelet aggregations. LQ represent the patient’s quartile with the lowest baseline epinephrine induced platelet aggregations while UQ is patient’s quartile with the highest values.**

Normally distributed parameters are given in a mean ± standard error of the mean (SEM) format, parameters with non-normal distributions were shown as median and interquartile ranges (IQR). Categorical values are given as n and percentage of the corresponding group (%).

Abbreviations: BMI: body mass index, BP: blood pressure, CAD: coronary artery disease, AMI: acute myocardial infarction, PCI: percutaneous coronary intervention, PAD: peripheral artery disease, STEMI: ST-segment elevation myocardial infarction, NSTEMI: non ST-segment elevation myocardial infarction, UAP: unstable angina pectoris, LM: left main coronary artery, LAD: left anterior descending artery, LCX: left circumflex artery, RCA: right coronary artery, DES: drug eluting stent, MACE: major adverse cardiovascular event, AE: adverse event, CV: cardiovascular, CABG: coronary artery bypass grafting, ER: emergency room, CCS: Canadian Cardiovascular Society angina grade, NYHA: New York Heart Association functional classification.

**Supplementary Table 2.**

| **Medical therapy** | **Total cohort (n=75)** | **LQ (n=19)** | **Q2 (n=19)** | **Q3 (n=18)** | **UQ (n=19)** |
| --- | --- | --- | --- | --- | --- |
| **Antiplatelet and anticoagulant therapy** | |  |  |  |  |
| **ASA, 100mg**  at baseline, (%)  at 3 months, (%)  at 12months, (%) | 98  98  98 | 100  100  100 | 100  100  100 | 100  100  100 | 95  95  95 |
| **Clopidogrel 75mg/150mg**  at baseline, (%)  at 3 months, (%)  at 12 months, (%) | 78/7  80/0  75/0 | 85/10  90/0  92/0 | 79/5  74/0  62/0 | 72/0  72/0  73/0 | 80/10  84/0  76/0 |
| **Prasugrel, 10mg**  at baseline, (%)  at 3 months, (%)  at 12 months, (%) | 13  17  10 | 5  10  0 | 16  21  15 | 28  28  18 | 5  5  6 |
| **Ticagrelor, 90mg**  at baseline, (%)  at 3 months, (%)  at 12 months, (%) | 2  2  2 | 0  0  0 | 0  0  0 | 0  0  0 | 5  11  6 |
| **No P2Y_12_ inhibitor**  at baseline, (%)  at 3 months, (%)  at 12 months, (%) | 0  1  13 | 0  0  8 | 0  5  23 | 0  0  9 | 0  0  12 |
| **VKA**  at baseline, (%)  at 3 months, (%)  at 12 months, (%) | 11  11  11 | 11  5  5 | 22  22  22 | 11  5  5 | 11  5  5 |
| **Medical therapy at enrolment** | |  |  |  |  |
| ACE-inhibitor/ARB, (%) | 93/5 | 89/11 | 100/0 | 100/0 | 95/5 |
| Beta –blocker, (%) | 97 | 100 | 89 | 100 | 100 |
| CCB, (%) | 5 | 0 | 0 | 11 | 11 |
| Nitrates, (%) | 19 | 32 | 11 | 17 | 16 |
| MRA, (%) | 16 | 21 | 5 | 17 | 26 |
| OAD/insulin, (%) | 15/3 | 11/0 | 11/0 | 28/6 | 11/5 |
| PPI, (%) | 88 | 90 | 79 | 94 | 90 |
| Diuretics, (%) | 16 | 16 | 11 | 11 | 5 |
| Statin,  at baseline, (%)  at 3 months, (%)  at 12 months, (%) | 97  97  89 | 95  95  85 | 100  100  92 | 100  94  91 | 95  100  88 |

**Supplementary Table 2. Antiplatelet/anticoagulant therapy throughout the study period and conventional medical therapy at enrolment in ACS patients with the four subgroups according to baseline epinephrine induced platelet aggregations. LQ represent the patient’s quartile with the lowest baseline epinephrine induced platelet aggregations while UQ is patient’s quartile with the highest values.**

Values are given as percentage of the corresponding total groups.

Abbreviations: ASA: acetylsalicylic acid, VKA: vitamin K antagonist, ACE: angiotensin converting enzyme, ARB: angiotensin receptor blocker, CCB: calcium channel blocker, MRA: mineralocorticoid receptor antagonist, OAD: oral antidiabetic therapy, PPI: proton-pump inhibitor.

**Supplementary Table 3.**

| **Laboratory parameters** | **Total cohort** | **LQ** | **Q2** | **Q3** | **UQ** |  |
| --- | --- | --- | --- | --- | --- | --- |
| Na (mmol/l) | 142 (141-143) | 141 (140-143) | 142 (141-143) | 142 (141-143) | 142 (141-143) | |
| K (mmol/l) | 4.29 (4.06-4.52) | 4.1 (4.0-4.37) | 4.2 (4.0-4.4) | 4.4 (4.1-4.7) | 4.3 (4.1-4.6) | |
| CN (mmol/l) | 5.8 (5.1-6.6) | 5.4 (4.5-6.1) | 6.2 (5.0-6.9) | 5.9 (5.2-6.6) | 5.8 (5.0-6.7) | |
| Creatinine (µmol/l) | 87 (75-94) | 91 (78.0-97.0) | 84 (66-94) | 79 (67-88) | 87.0 (78.0-99.0) | |
| Bilirubin (µmol/l) | 10.35 (8.2-12.45) | 9.3 (7.85-10.95) | 11.5 (9.2-15.8) | 9.5 (7.2-12.4) | **10.2 (7.9-14.6)** | |
| GGT (U/l) | 29 (21-48.5) | 30.5 (20.5-38.5) | 23 (20-40) | 27 (23-39) | **41.5 (21.5-77.5)** | |
| ALP (U/l) | 80.5 (63-94) | 86.5 (67.5-94.5) | 82 (62-107) | 70.5 (57.5-82) | 84.0 (70.5-94.0) | |
| TSH (µIU/ml) | 1.43 (0.91-2.15) | 0.95 (0.81-1.89) | 1.63 (1.02-2.47) | 1.47 (1.29-2.63) | **1.49 (1.19-1.86)** | |
| Red blood cell count (T/l) | 4.66 (4.41-4.86) | 4.72 (4.56-4.86) | 4.74 (4.48-4.94) | 4.55 (4.34-4.75) | 4.65 (4.35-4.83) | |
| Haemoglobin (g/l) | 139 (130-147) | 145 (136-149) | 139 (131-150) | 136 (127-144) | 138 (133-145) | |
| Haematocrit (L/l) | 0.41 (0.39-0.43) | 0.42 (0.41-0.43) | 0.41 (0.39-0.43) | 0.39 (0.37-0.42) | 0.40 (0.39-0.42) | |
| Platelet count (G/l) | 211 (184-247) | 198 (184-291) | 211 (180-245) | 204 (193-262) | 216 (181-229) | |
| Mean platelet volume (fl)  on admission  at enrolment  at 3 months  at 12 months | 10.5 (10.0-11.0)  10.7 (10.3-11.3) Ł  10.8 (10.3-11.4)  10.5 (10-11.3) | 10.4 (9.9-10.8)  10.7 (10.3-11.2)  10.4 (10.3-11.1)  10.5 (10.0-11.1) | 10.9 (10.2-11.3)  11.1 (10.5-11.3)  10.9 (10.6-11.5)  10.5 (10.1-11.4) | 10.3 (9.7-11.1)  10.6 (10.0-11.1)  10.9 (10.3-11.2)  10.5 (10.1-11.3) | 10.7 (9.9-11.0)  10.6 (10.3-11.3) Ł  10.6 (10.2-11.2)  10.6 (10.0-11.4) | |
| **Cardiac biomarkers** |  |  |  |  |  | |
| hsTroponin T (ng/ml) |  |  |  |  |  | |
| on admission  at enrolment | 384.8 (112.2-1497)  963.5 (319.1-2146) | 199.4 (15.9-629.8)  831.1 (152.0-1258.0) | 337.8 (124.1-641.2)  942.3 (389.2-1767.0) | 669.8 (219.6-2904.0)  2117.5 (319.1-3934.0) | **669.3 (175-2544) $**  **837.8 (347.5-2283.0)** | |
| CK (U/l) |  |  |  |  |  | |
| on admission  at enrolment  at 3 months  at 12 months | 304 (129-684)  149 (105-245)  122 (90-167)  118 (97-204) | 201 (117-676)  148 (94-234)  134 (108-191)  107 (96-195) | 146 (104-188)  247 (165-379)  122 (87.157)  118 (101-225) | 530 (211-1651)  175 (110-257)  109 (86-127)  124 (116-170) | 380 (118-1261)  170 (85-331)  132 (90-199)  121 (94-269) | |
| CK-MB (U/l) |  |  |  |  |  | |
| on admission  at enrolment | 11.9 (4.7-71.4)  3.5 (2.2-5.7 | 6.2 (3.3-91.1)  3.11 (2.2-4.7) | 14.2 (4.5-37.8)  3.6 (2.2-7.3) | 27.2 (7.3-178.5)  3.9 (3.2-6.9) | **22.1 (4.8-92.1)**  2.9 (1.9-5.4) | |
| ASAT (U/l)  on admission  at enrolment  at 3 months  at 12 months | 41 (28-69)  29 (22-44)  22 (18-27)  22 (18-27) | 29 (20-58)  22 (20-31)  22 (18-28)  20 (19-30) | 37 (30-58)  28 (19-50)  23 (18-27)  22 (17-23) | 53 (26-138)  31 (24-46)  21 (19-27)  23 (19-27) | 63 (30-92)***  **37 (26-50)**  23 (18-27)  23 (18-25) | |
| ALAT (U/l) |  |  |  |  |  | |
| on admission  at enrolment  at 3 months  at 12 months | 30 (23-45)  29 (21-42)  27 (18-37)  25 (18-30) | 23 (18-38)  28 (18-33)  29 (25-38)  24 (21-29) | 27 (22-36)  27 (19-50)  27 (15-34)  24 (17-27) | 31 (26-51)  31 (24-46)  26 (19-33)  26 (21-30) | 43 (26-61)***  42 (22-62)***  26 (17-38)  29 (18-33) | |
| LDH (U/l) |  |  |  |  |  | |
| on admission  at enrolment  at 3 months  at 12 months | 383 (329-626)  489 (380-708)  197 (180-220)  196 (174-211) | 350 (263-400)  479 (345-558)  186 (167-228)  200 (165-208) | 435 (367-457)  516 (318-626)  208 (184-225)  201 (163-229) | 401 (361-912)  641 (418-1045)  189 (175-202)  196 (186-224) | 466 (360-693)***  **493 (404-775)**  **204 (186-235)**  193 (176-206) | |
| **Inflammatory markers** |  |  |  |  |  | |
| CRP (mg/l) |  |  |  |  |  | |
| on admission,  at enrolment,  at 3 months,  at 12 months, | 3.36 (1.06-10.8)  14.12 (5.39-34.4)^+^  1.76 (0.61-3.92)  0.98 (0.6-2.67) | 2.68 (0.32-11.56)  15.27 (2.17-26.14)^+^  1.54 (0.60-3.52)  0.98 (0,60-1,08) | 4.2 (0.82-10.6)  13.9 (5.7-22.6)  2.33 (0.62-8.64)  1.69 (0.69-2.67) | 2.6 (0.68-7.34)  14.3 (6.39-38.0)  1.57 (0.98-2.45)  0.71 (0.60-2.32) | **4.67 (2.74-23.3)**  **12.30 (5.02-50.49)^+^**  1.52 (0.60-4.32)  0.98 (0.62-3.43) | |
| Leukocyte count (G/l) |  |  |  |  |  | |
| on admission,  at enrolment,  at 3 months,  at 12 months, | 9.8 (8.2-13.1)*  8.0 (6.6-9.4)  6.8 (5.7-8.7)  6.7 (5.4-7.7) | 9.4 (8.2-11.4)*  8.2 (6.9-9.8)  6.9 (5.8-8.1)  6.7 (5.8-7.3) | 9.8 (8.8-13.3)  8.2 (6.8-10.1)  7.2 (5.7-8.8)  6.8 (5.3-8.1) | 9.4 (7.6-11.6)  7.1 (6.5-9.3)  6.2 (5.7-7.9)  6.0 (5.5-8.4) | 10.8 (6.9-13.2)*  7.6 (6.1-9.2)  6.5 (5.7-8.9)  6.37 (5.9-7.4) | |
| **Lipid parameters** |  |  |  |  |  | |
| Total cholesterol (mmol/l) |  |  |  |  |  | |
| on admission,  at 3 months,  at 12 months, | 5.0 (4.0-5.5)  3.4 (3.0-4.1) &  3.6 (3.0-4.4) & | 5.0 (4.0-5.3)  3.5 (3.1-3.6) &  **3.4 (3.2-4.0)** | 4.9 (4.1-6.0)  3.3 (2.7-4.7)  3.4 (3.3-4.2) | 5.1 (4.2-5.4)  3.4 (3.0-3.7)  3,1 (2.8-3.7) | 5.0 (3.8-5.4)  3.3 (2.9-4.1) &  **4.0 (3.1-5.0)** | |
| LDL-cholesterol (mmol/l) |  |  |  |  |  | |
| on admission,  at 3 months,  at 12 months, | 3.0 (2.4-3.8)  1.7 (1.4-2.4) &  1.8 (1.4-2.5) & | 2.9 (2.4- 3.1)  1.7 (1.5-2.2) &  1.8 (1.4-2.1) & | 2.9 (2.3-3.8)  1.7 (1.3-3.0)  1.8 (1.5-2.3) | 3.2 (2.7-3.8)  1.8 (1.4-2.3)  1.6 (1.0-2.0) | 3.2 (1.8-4.1)  1.7 (1.2-2.5) &  2.1 (1.4-2.6) & | |
| HDL-cholesterol (mmol/l) |  |  |  |  |  | |
| on admission,  at 3 months,  at 12 months, | 1.1 (1.0-1.3)  1.0 (0.9-1.2) &  1.1 (1.0-1.3) | 1.2 (1.0-1.3)  1.0 (0.8-1.2) &  1.2 (1.0-1.3) | 1.2 (1.0-1.4)  1.1 (1.0-1.2)  1.1 (1.0-1.4) | 1.1 (1.0-1.3)  1.0 (0.9-1.2)  1.1 (0.9-1.3) | 1.1 (1.1-1.3)  1.0 (0.9-1.2)  1.1 (0.9-1.2) | |
| Triglyceride (mmol/l) |  |  |  |  |  | |
| on admission,  at 3 months,  at 12 months, | 1.5 (1.2-1.9)  1.2 (1.0-1.7) &  1.2 (0.9-2.2) | 1.8 (1.2-1.9)  1.2 (1.0-1.6)  1.3 (1.0-1.4) | 1.5 (1.1-2.1)  1.2 (0.7-1.7)  1.1 (0.8-1.5) | 1.2 (1.1-1.6)  1.2 (0.9-1.7)  1.1 (0.9-2.1) | 1.6 (1.2-2.5)  1.3 (1.0-2.0) &  1.6 (1.0-2.4) | |
| **Salivary cortisol levels** | |  |  |  |  | |
| at enrolment,  at 3 months,  at 12 months, | 11.0 (8.0-14.2)  11.3 (7.65-14.6)  7.8 (5.45-12.85)** | 9.4 (7.8-12.2)  10.5 (9.0-13.3)  6.8 (4.45-13.5) | 12.0 (7.9-20.1)  14.1 (7.1-20.6)  9.1 (6.8-12.5) | 11.5 (6.6-14.4)  7.9 (5.7-11.4)  6.4 (5.1-9.9) | **11.5 (8.9-13.5)**  **12.1 (7.8-14.9)**  **8.5 (5.4-13.7)** | |

**Supplementary Table 3. Laboratory parameters, cardiac and inflammatory biomarkers and salivary cortisol levels upon admission, at enrolment (96 hours) and at 3- and 12-months follow-up in the total cohort and in the four subgroups according to baseline epinephrine induced platelet aggregations. LQ represent the patient’s quartile with the lowest baseline epinephrine induced platelet aggregations while UQ is patient’s quartile with the highest values.**

Values are medians ± lower and upper quartile ranges. Numbers in bold indicate that the parameters are higher in the upper quartile group, but the difference did not reach the statistical significance (Mann-Whitney tests, n=19).

.Ł: MPV values are higher at enrolment than on admission, p<0.05, for the LQ group p=0.07.

$ p=0.07, n=19, Mann-Whitney test between troponin levels on admission in lower and upper quartile patients.

*** p<0.05 ASAT, ALAT and LDH levels are higher in the upper quartile group.

+: CRP levels are significantly lower on admission than at enrolment with p values are <0.05 in the total cohort and the lower quartile patient group. p=0.058 in the upper quartile patients (Wilcoxon matched pairs test).

*leukocyte count was significantly higher (p<0.05) on admission compared to enrolment or 3 and 12 months controls.

&: p<0.05 Wilcoxon matched pairs test between on admission and 3 or 12 months values,

**­ indicate that salivary cortisol levels at 12 months are significantly reduced compared to baseline and to 3 months follow-up, p<0.05.

Abbreviations: Na: sodium; K: potassium; CN: carbamide; GGT: gamma-glutamyl transferase; ALP: alkaline phosphatase; TSH: thyroid-stimulating hormone; CK: creatin kinase, CK–MB: creatin kinase muscle/brain isoform, ASAT: aspartate aminotransferase; ALAT: alanine aminotransferase, LDH: lactat dehydrogenase, CRP: C-reactive protein; HDL: high-density lipoprotein; LDL: low-density lipoprotein.

**Supplementary Table 4.**

| **Medical therapy at enrolment, 3 months and 12 months follow-ups** | | |
| --- | --- | --- |
|  | **Number of treated patients in the lower quartile epinephrine induced aggregations subgroup**  **At enrolment/3 months/12 months** | **Number of treated patients in the upper quartile epinephrine induced aggregations subgroup**  **At enrolment/3 months/12 months** |
| **ACE inhibitors** |  |  |
| **Enalapril, n** |  |  |
| 1x2.5mg | 0/0/1 | 0/0/1 |
| 2x2.5 mg | 3/2/0 | 3/3/3 |
| 2x5 mg | 6/5/4 | 4/3/1 |
| 2x7.5mg | 0/0/0 | 1/0/0 |
| 2x10mg | 2/1/1 | 3/3/3 |
| 2x15mg | 0/2/2 | 2/0/0 |
| 2x20mg | 1/0/1 | 0/1/1 |
| **Perindopril, n** |  |  |
| 1x5mg | 1/3/4 | 2/4/3 |
| 1x8mg | 1/0/0 | 0/0/0 |
| 1x10mg | 1/0/1 | 0/1/1 |
| **Ramipril, n** |  |  |
| 1x5mg or 2x2.5mg | 2/1/0 | 1/2/2 |
| 2x5mg | 0/2/0 | 1/0/0 |
| **Fosinopril, n** |  |  |
| 1x10mg | 1/0/0 | 0/1/0 |
| **ARB** |  |  |
| **Telmisartan, n** |  |  |
| 1x80mg | 0/1/0 | 0/0/0 |
| 1x150mg | 0/0/1 | 0/0/0 |
| **Irbesartan, n** |  |  |
| 1x150mg | 0/0/1 | 0/0/0 |
| 1x300mg | 1/1/0 | 1/0/1 |
| **Valsartan, n** |  |  |
| 2x80mg | 0/1/0 | 0/0/1 |
| 2x160mg | 0/0/1 | 1/0/0 |
| **No ACEI/ARB, n** | 0/0/2 | 0/1/2 |
| **MRA** |  |  |
| **Eplerenone, n** |  |  |
| 1x25 mg | 1/1/1 | 2/2/1 |
| 1x50mg | 1/1/1 | 1/1/2 |
| **Spironolactone, n** |  |  |
| 1x12.mg | 0/0/0 | 1/1/1 |
| 1x25mg | 1/1/1 | 0/0/1 |
| 1x50mg | 1/1/1 | 1/1/1 |
| **Beta blockers** |  |  |
| **Bisoprolol, n** |  |  |
| 1x1.25mg | 5/3/3 | 2/1/1 |
| 1x2.5mg | 8/7/9 | 9/8/9 |
| 1x5mg or 2x2.5mg | 2/5/2 | 3/5/4 |
| 2x5mg or 1x10mg | 1/3/3 | 1/4/3 |
| **Metoprolol, n** |  |  |
| 2x12.5mg | 1/0/0 | 0/0/0 |
| 2x25mg | 0/1/1 | 1/0/0 |
| 2x75mg | 1/0/0 | 0/0/0 |
| **Nebivolol, n** |  |  |
| 1x2.5mg | 0/0/0 | 1/0/1 |
| 1x5mg | 1/0/0 | 0/0/0 |
| **Carvedilol, n** |  |  |
| 2x12.5mg | 0/0/0 | 1/1/1 |
| **No beta blockers, n** | 0/0/1 | 0/0/0 |

**Supplementary Table 4. Medical therapy affecting the neurohormonal system in ACS patient subgroups with lower and upper quartile epinephrine induced aggregations. Number of treated patients are provided at enrolment, at 3- and 12-months follow-ups for patient groups with high or low platelet adrenergic activity. Total patient number is 19 in each subgroup.**

**Supplementary Table 5.**

| Baseline aggregations | STEMI | NSTEMI | p |
| --- | --- | --- | --- |
| 1 μg/ml collagen (mean ± SD) | 25.7 ± 22.7% | 25.8 ± 23.0% | 0.89 |
| 2 μg/ml collagen (mean ± SD) | 41.8 ± 23.1% | 38.2 ± 27.9% | 0.33 |
| 1.25 μM APD (mean ± SD) | 33.2 ± 21.9% | 32.3 ± 19.8% | 0.93 |
| 5 μM ADP (mean ± SD) | 49.2 ± 18.6% | 45.7 ± 18.8% | 0.46 |
| 10 μM ADP (mean ± SD) | 48.3 ± 19.5% | 50.7 ± 19.2 % | 0.49 |
| 2μg/ml epinephrine (mean ± SD) | 41.4 ± 22.3% | 37.5 ± 27.3% | 0.36 |
| 0.5 μg/ml arachidonic acid (mean ± SD) | 5.5 ± 9.8% | 6.2 ± 13.5 % | 0.55 |

**Supplementary Table 5. Subgroup analysis of STEMI and NSTEMI patients indicating no statistical difference between initial platelet aggregation values. Aggregations were measured at enrolment, 96 hours after hospital admission on dual antiplatelet therapy.**
